# An experimental method to identify neurogenic and myogenic active mechanical states of intestinal motility

**DOI:** 10.3389/fnsys.2013.00007

**Published:** 2013-04-11

**Authors:** Marcello Costa, Lukasz Wiklendt, John W. Arkwright, Nicholas J. Spencer, Taher Omari, Simon J. H. Brookes, Phil G. Dinning

**Affiliations:** ^1^Department of Human Physiology, School of Medicine, Flinders UniversityBedford Park, SA, South Australia; ^2^Department of Medicine, St. George Clinical School, University of New South WalesKogarah, NSW, Australia; ^3^Material Science and Engineering, CSIROSydney, NSW, Australia; ^4^Gastroenterology Unit, Child, Youth and Women's Health ServiceNorth Adelaide, SA, Australia; ^5^Department of Gastroenterology and Surgery, Flinders Medical CentreAdelaide, SA, Australia

**Keywords:** intestinal motility, enteric neurons, intestine biomechanics, manometry, spatio-temporal maps, rabbit colon

## Abstract

Excitatory and inhibitory enteric neural input to intestinal muscle acting on ongoing myogenic activity determines the rich repertoire of motor patterns involved in digestive function. The enteric neural activity cannot yet be established during movement of intact intestine *in vivo* or *in vitro*. We propose the hypothesis that is possible to deduce indirectly, but reliably, the state of activation of the enteric neural input to the muscle from measurements of the mechanical state of the intestinal muscle. The fundamental biomechanical model on which our hypothesis is based is the “three-element model” proposed by Hill. Our strategy is based on simultaneous video recording of changes in diameters and intraluminal pressure with a fiber-optic manometry in isolated segments of rabbit colon. We created a composite spatiotemporal map (DPMap) from diameter (DMap) and pressure changes (PMaps). In this composite map rhythmic myogenic motor patterns can readily be distinguished from the distension induced neural peristaltic contractions. Plotting the diameter changes against corresponding pressure changes at each location of the segment, generates “orbits” that represent the state of the muscle according to its ability to contract or relax actively or undergoing passive changes. With a software developed in MatLab, we identified twelve possible discrete mechanical states and plotted them showing where the intestine actively contracted and relaxed isometrically, auxotonically or isotonically, as well as where passive changes occurred or was quiescent. Clustering all discrete active contractions and relaxations states generated for the first time a spatio-temporal map of where enteric excitatory and inhibitory neural input to the muscle occurs during physiological movements. Recording internal diameter by an impedance probe proved equivalent to measuring external diameter, making possible to further develop similar strategy *in vivo* and humans.

## Introduction and background

Progression of intestinal contents along the digestive tube is due to the coordinated contractions and relaxations of the smooth muscle layers. Intestinal smooth muscles are controlled by myogenic mechanisms (initiated by non-neural pacemaker cells) and neurogenic mechanisms (mediated by enteric neurons), which interact to generate the diverse motor behavior of the intestine (Box 1). Studies of the movements of the intestinal wall and of its contents are based on the ability to record mechanical features of the intestinal wall, due to activity of the intestinal muscle.

Box 1 **Principles of motor control by ENS; the intestinal muscular apparatus and the enteric nervous system.**

The mechanical state of the intestinal muscle activity is controlled by two main parallel but interacting mechanisms. One broadly referred to as myogenic, consists of a network of pacemaker cell called ICC, pacemaker cells and these generate rhythmic oscillations of muscle membrane potential, which, when they reach a electrical threshold, result in phasic contractions. Superimposed on this myogenic activity, complex circuits within the enteric nervous system (ENS) determine different motor patterns, including spontaneous slow migrating motor complexes (MMCs), content dependent peristaltic contractions and neural accommodation. *In vivo* the motor functions of the digestive tract are also modulated by extrinsic nerves.

**The intestinal muscle**

The intestinal smooth muscle layers are organized as a syncitium of individual smooth muscle cells connected electrically via gap junctions. The smooth muscle is capable both of active contractions and active relaxations. Contractions are due to entry, at particular threshold, of Ca ions via voltage dependent channels (L-type Ca2). Depolarization of the muscle can activate direct excitation-contraction coupling and in many instances can trigger Ca2 action potentials. The action potentials propagate poorly across the gap junctions between muscle cells and the passive decay in the amplitude of the currents leads to relatively localized, non-propagating, areas of contraction. Relaxation after contraction occurs passively by reduction in intracellular Ca2 to basal levels by uptake in intracellular stores or by extrusion from the cell. Active relaxations are due to the activation of intracellular cGMP resulting in further reduction in intracellular calcium, etc.

**Sources of depolarization in smooth muscle**

Smooth muscle can be depolarized by pacemaker cells or by enteric neurons.

Spontaneous oscillations in the membrane potential of the interstitial cells of Cajal (ICCs) drive the adjacent intestinal smooth muscle cells to oscillate (slow waves). The ICCs are interconnected by gap junctions and each ICC can influence and entrain the adjacent ICCs so that groups of ICCs can oscillate in synchrony. If there is a gradient in the intrinsic frequencies of the ICCs, the ones with faster rates will entrain the slower ones. Since there is a delay in the coupling across the gap junctions between ICCs, this will generate active, non-decremental propagation of the oscillations. This will result in a parallel propagation of slow waves in the passively driven smooth muscle cells, with frequency, direction, and speed of propagation being determined by the oscillations within the pacemaker net.

There are at least two pacemaker nets in the mammalian digestive tract, one at the level of the myenteric plexus (ICC-MY) and one at the junction between circular muscle and submucosa (ICC-SM). This distinction is more pronounced in the large intestine than in the small intestine or the stomach, where the main pacemaker net is the ICC-MY.

As the smooth muscle is under the ongoing influence of the pacemaker nets, the term *muscular apparatus* refers conjointly to the muscle and pacemaker cells. Myogenic activity refers to the spontaneous activity of the muscular apparatus.

**Excitation and inhibition of intestinal smooth muscle by enteric neurons**

Enteric neurons form circuits within the intestinal wall (ENS) involved in motor, secretory, and circulatory functions. All motor output from these enteric circuits is channeled through the final excitatory and inhibitory motor neurons. Excitatory enteric neurons depolarize smooth muscles cells directly via the release of excitatory transmitters, acetylcholine and tachykinins on the muscle directly, or indirectly via specialized ICCs. The transient nerve mediated depolarizations in the smooth muscle are described as excitatory junction potentials (EJPs).

Enteric inhibitory motor neurons mediate inhibition by hyperpolarizing the smooth muscle directly via NO or ATP or by inhibiting the contraction-coupling apparatus via VIP or indirectly via specialized ICCs. The transient nerve mediated hyperpolarizations in the smooth muscle are described as inhibitory junction potentials (IJPs).

**Patterns of intestinal motility by interplay of myogenic and neurogenic mechanisms**

Myogenic mechanisms generate ongoing spontaneous rhythmic activity, which at a mechanical threshold can result in motor activity with frequency, direction and speed of propagation of contractions determined by the pacemaker nets.

Neurogenic mechanisms refer to the activity of enteric circuits resulting in specific motor patterns.

Example of clear myogenic motor pattern is the antral peristalsis driven by the gastric ICCs nets, which in normal conditions show a clear gradient from corpus to pylorus, ensuring aboral propagation of contractions responsible for gastric emptying. However gastric slow waves only result in effective propulsive contractions if neural inputs, usually from excitatory enteric neurons activated by vagal inputs, are superimposed on the muscular apparatus. This is an example of a permissive role of neural circuits on gastrointestinal motor patterns. Another clear example of myogenic patterns emerging as mechanically effective is the propagating circular muscle contractions in the small intestine during the phase III of the MMCs, when every slow wave over a length of intestine, are driven by the activity of excitatory enteric neurons. The result are “myogenic peristaltic contractions,” in which their frequency, direction ad speed are determined by the pacemaker nets of ICCs.

In most other instances slow waves by themselves produce little, and often no mechanical activity.

All other patterns of motor activity that organize gastrointestinal movements are organized by specific activation of enteric neural circuits (Neurogenic mechanisms). Such patterns include the migrating myoelectric (motor) complexes in the small intestine (MMCs), which initiate proximally, often in the stomach, and migrate to the distal small intestine, recurring cyclically at long interval even, and particularly in the absence of food content in the intestine (interdigestive events). Whether in the colon similar spontaneous neurogenic motor activity occurs, described as CMMCs, or whether the ENS only plays a permissive role on the underlying myogenic processes remains to be established.

The propulsive motor patterns that is elicited by distension or by chemical contents, described classically as “peristalsis,” is mediated by enteric circuits involving polarized enteric reflex pathways, i.e., ascending excitatory, descending inhibitory ad possibly descending excitatory pathways. Despite such pathways can be activated by localized stimuli, and thus described as polarized enteric reflexes, the actual propulsion cannot be regarded as a simple “reflex” (the term peristaltic reflex has been often used), but rather a neurally coordinated behavior. As this motor pattern is initiated by luminal contents and require polarized enteric circuits, is currently referred to as “neural peristalsis.” Accommodation of luminal contents involves enteric inhibitory “reflex” pathways and operate physiologically in the stomach, small and large intestine to allow filling before emptying. Retrograde peristalsis (antiperistalsis) describes potentially neurogenic and myogenic patterns and these have not been elucidated well.

All these “neurogenic” motor patterns, act on the ongoing spontaneous myogenic activities and thus the final mechanical events are always the result of the interplay between these two main mechanisms.

The intestinal wall is composed of several layers including smooth muscle, mucosa, and connective tissue. From a mechanical point of view the intestinal wall can be regarded as a viscoelastic body (Meiss, 2011). The “three-element model” proposed by Hill (1938, 1970) considers the tissue to be composed of a “contractile element,” responsible for the “active component” of the muscle, connected with a “series elastic element,” and a “parallel element” to describe the connective tissue. The parallel elastic component seems to consist of the connective tissue as the contractile element of smooth muscle which by itself does not contribute significantly to the passive tension (Weems, 1981; Fung, 1993; Gregersen and Kassab, 1996; Gregersen, 2002; Nicosia and Brasseur, 2002; Gregersen et al., 2009; Meiss, 2011).

While in principle the mechanical behavior of the digestive tube can be described in terms of mechanical properties of visco-elastic materials and in terms of dynamic fluid mechanics, significant difficulties have hindered development of useful models to understand the mechanical factors of intestinal motility. Intestinal motility is complicated by the fact that the pump supplying energy and the conduit that contains the propelled content are the same organ, unlike, for example, the heart and vascular system. The relationships between wall motion, pressure and flow in the intestine require complex coupled mathematical equations and hence remain poorly defined.

One major challenge for neurogastroenterology is to identify the patterns of activation of enteric motor neural circuits during intestinal movements. This would require recording of the activity of enteric neurons with either intracellular recording (Spencer and Smith, 2004) or voltage dyes (Mazzuoli and Schemann, 2009). Neither method can be applied at present to the intact moving intestine. The enteric neurons are located within the gut wall and cannot be accessed in the intact intestine. Furthermore intracellular recordings require stability of the tissue, and voltage dyes require direct visual access.

The strategy developed in this study is to infer activity of enteric neurons and pacemaker cells from the mechanical state of the muscle established by simultaneous measures of changes in diameter of the gut wall and underlying intraluminal pressure (Box 1). The beauty of our strategy is twofold; (1) Recording both changes in intraluminal pressure and diameter is readily achievable, using a relatively simple setup; and (2) These data can be recorded from long sections of gut (10–30 cm) thereby allowing the researcher to simultaneously define the differing mechanical states over the entire lengths of the preparation at any single point in time or all points in time.

In physiological conditions, excitation of smooth muscle, by myogenic and/or neurogenic mechanisms will cause either shortening, or increased tone, or a combination of the two, depending on the resistance that the muscle is working against. If resistance is high, muscle excitation will lead to increased tone (and increased pressure) but little shortening, i.e.: an isometric contraction. If resistance is negligible, the muscle will shorten nearly isotonically and little pressure (or wall stress) will be generated. In a gut full of viscous fluid content, the extent of local resistance to contraction will be determined by how readily content can flow axially. Thus in most cases, local excitation of muscle will lead to an “auxotonic” contraction, defined as combination of shortening and increased muscular stress (correlated with locally increased intraluminal pressure). Thus excitation of muscle, by myogenic and/or neurogenic mechanisms can be considered as a reversible change in compliance/stiffness of a visco-elastic muscle.

Conversely, removal of excitation, or active neurogenic inhibition of a muscle (Waterman et al., 1994) can be conceptualized as a temporary, reversible increase in compliance. Whether this leads to either increased length depends on the availability of viscous fluid content from neighboring regions. The interplay of excitatory and inhibitory neurogenic input with myogenic mechanisms and their resulting effects on the distribution of content generates the rich repertoire of intestinal motor patterns (Box 2).

Box 2 **Principles of biomechanical properties of the intestine.**

The intestinal wall is composed of several layers including smooth muscle, mucosa, and connective tissue, and collectively these can be regarded as a viscoelastic body (Meiss, 2011). The mechanical properties of this body have been described by Hill (1938, 1970) as a “three-element model” in which the tissue is composed of a “contractile element,” responsible for the “active component” of the muscle, connected with a “series elastic element,” and a “parallel element” to describe the connective tissue.

To define various mechanical states of the intestinal tissue the following relations are commonly used;

1. *Length/tension curves*; define the relation between length of a strip of intestine relative to resting length and the relative *tension* of the tissue.
2. Stress/strain curve; defines the force that acts on the intestinal strip over a cross sectional area. In such a relation *stress* is the force applied per unit cross-sectional area and *strain* describes the mechanical deformation of the tissue.
3. From the length/tension or stress/strain curves, “active stress,” generated by the unique contractile property of muscle cells, can be distinguished from “passive stress,” which arises from the passive physical properties of the tissue (Gordon and Siegman, 1971a,b; Fung, 1993; Gregersen and Christensen, 2000). Thus is possible to estimate the contractile (active) state of the muscle. The contractile state of the smooth muscle is the main determinant of the tension of the intestinal wall within the normal range of diameters. Only at greater distension is the tension of the wall determined by the passive properties of the tissue (see Figures; Fung, 1993).
4. The original idea of Hill regarding the “active component” of the muscle, intuitively still used as “contraction” or “contractile state,” has given way to the concept of muscle tone. Tone may be thus synonymous with the “active stress” generated in the muscle wall (Ghosh et al., 2005).

**Mechanical states**

Equivalent mechanical states of the intestinal wall can be recorded in isolated strips and correspondingly in tubular preparations of intestine.

- *Isometric contraction*; in muscle strips occur when the load is greater than the force of contraction and this results in no change to the length of the muscle strip.
- *Isovolumetric contraction*; in tubular preparations occurs when an intraluminal pressure is greater than the force of contraction. Hence there are changes in pressure but not in diameter.
- *Isotonic contraction*; in muscle strips occur as the force of the muscle becomes greater than the load, lifting that load and generating work.
- *Isobaric contraction*; in tubular preparations occurs when the muscle force exceeds intraluminal pressure, the circular muscle shortens with a decrease in diameter.
- *Auxotonic contraction*; occurs when the muscle changes force and length simultaneously.

The intestinal muscle can also be inhibited actively by relaxing pharmacological agents and by enteric inhibitory motor neurons. The corresponding active states can be described as

- *Isometric relaxation* in strips and *isovolumetric relaxations* in tubular preparations.
- *Isotonic relaxation* in strips and *isobaric relaxations* in tubular preparations.
- *Auxotonic relaxation* in both strips and tubular preparations.

Because there is a mechanical equivalence of strip and tubular preparations, with the muscle circumferential tangential force calculated by Laplace's law, we will use the terms isometric, isotonic and auxotonic for all recordings.

The force measured as a force (isometric) is also a measure of tension (force acting on a material) or load. The mechanical description is the same, whether the force is exerted on the tissue by an imposed strain from outside or is generated by an intrinsic force from the contractile system of the muscle. This reflects the third law of Newton mechanics which defines two equal forces coming from opposite directions.

In the intact intestine changes in length of the circular muscle will result in changes in the circumference and thus the diameter of the lumen. Gut tissue is incompressible and therefore the reduction in diameter must be accompanied by concurrent changes in the wall thickness. This is the law of Mass conservation, in which the ratio of cross sectional area of the gut wall is equal to the ratio of the length of the rings of a given length (Nicosia et al., 2001). The contractions and relaxations and intestinal circular or longitudinal muscle, although are not attached to any fix point in the living organism, can be regarded as mechanical events of muscle strips attached to a lever with a variable load. As the circular muscle is usually thicker than the longitudinal muscle, when the two muscle layers contract simultaneously (Hennig et al., 1999; Spencer and Smith, 2001) under normal physiological conditions (Box 1), the circular muscle prevails with consequent passive elongation of the longitudinal axis.

In tubular preparations intraluminal pressure results from local tension of the intestinal wall (passive and active) acting on the luminal content (varying from gas to solid), as well as the force transmitted from other intestinal (or external) sites. The load in tubular preparations results from intraluminal pressure, generated by the forces acting on the luminal contents, that resists the shortening of the circular muscle.

*Laplace's law*; describes the relationship between the transmural pressure difference and the tension, radius, and thickness of the vessel wall. The higher the pressure difference the higher the circumferential tension, the thicker the wall the lower is the tension and the larger the diameter the greater the tension. These three rules culminate into one equation (modified Laplace's law; Nicosia and Brasseur, 2002). Tangential tension (Force) = (Pressure × radius)/wall thickness

Because the gastrointestinal tract is a reasonably uniform deformable cylinder, intestinal muscle tone can be defined as active stress tending to produce strain that leads to a reduction in luminal cross-sectional area and circumference (Gregersen et al., 1990; Ravi et al., 2010).

The relation between force and velocity of contraction, in both strip and tubular preparations, are governed by Hill's equation that describes a rectangular hyperbola. When the load on a muscle is less than its isometric tension-generating capability, the muscle shortens and lifts the load at a velocity that varies inversely with the size of the load.

A good indication of the state of intestinal muscle tone is the slope of the curve plotting changes in strain and stress, measured as compliance or its inverse stiffness. However the need to perform these measurements in static conditions, to take in account the passive viscoelastic properties of the intestine including stress relaxation, creep and hysteresis, makes meaningful measure of these parameters during movements of the intestine almost impossible (Gregersen, 2002; p. 67).

Here we propose to circumvent these difficulties by using the principles described in this brief summary and by doing so we hypothesize that it is possible to distinguish steady state conditions of intestinal muscle that correspond to active and passive changes in length and/or force.

Visualizing muscle excitation and inhibition as changes in compliance is conceptually useful, but experimentally frustrating. As Gregersen has described (Gregersen, 2002), compliance and stiffness cannot be quantified accurately during rapid dynamic intestinal movements, because such measurements needs quasi static conditions. In addition viscoelastic phenomena such as stress relaxation, creep and hysteresis are also impossible to analyse during ongoing motor activity. However, we believe that it is possible to deduce the ongoing level of excitation/inhibition of muscle at any point in the gut wall from the underlying intraluminal pressure and whether the muscle is shortening, or lengthening at any particular moment. This has become possible by combining several established techniques for recording gut motility. The first is spatiotemporal video diameter mapping (Hennig et al., 1999; Lentle et al., 2008; Huizinga et al., 2011). This technique measures the real-time wall motion of the gut at each point along a segment. The second technique is high resolution intraluminal pressure recording with a fiber-optic manometry catheter (Arkwright et al., 2009b). We have recently published a preliminary study successfully combining these techniques (Dinning et al., 2011). We explored a third technique, based on measurement of intraluminal impedance, which can give an indication of luminal cross section (from which internal diameter can be derived) to determine if we could reliably determine changes of internal diameters and thus, in combination with intraluminal pressure determine neural and myogenic activity in live human subjects, where external video recording is not feasible.

The present study was carried out on isolated segments of rabbit colon which shows robust myogenic and neurogenic motor patterns *in vitro* (Dinning et al., 2012a).

### The hypothesis

We propose to establish where and when active or passive contractions and relaxations of the intestinal muscle occur. This will enable us to map the intestinal motor patterns, in combination with the state of contractility of the intestinal muscle, and from this deduce where and when the final enteric excitatory and inhibitory motor neurons are active during intestinal movements.

## Methods

Eight New Zealand albino rabbits of both sexes weighing 2–4 kg were euthanized humanely by intravenous injection of sodium pentobarbitone (0.5 ml/kg) in accordance with approval by the Animal Welfare Committee of Flinders University. A ventral midline incision was made to expose the peritoneal cavity. Segments of the distal colon, 20–30 cm in length, were removed and placed immediately into beakers containing oxygenated Krebs solution (in mM: NaCl, 118; KCl, 4.7; NaH_2_PO4, 1.0; NaHCO_3_, 25; MgCl_2_, 1.2; D-Glucose, 11; CaCl_2:_ 2.5) bubbled with 95% O2/5% CO_2_. Fecal material was gently flushed out of the distal colonic segments. The rabbit distal colon was chosen because even in isolated segments all of the known myogenic and neurogenic patterns of motor activity are preserved (Dinning et al., 2012a).

### Experimental setup

**Figure 1:**
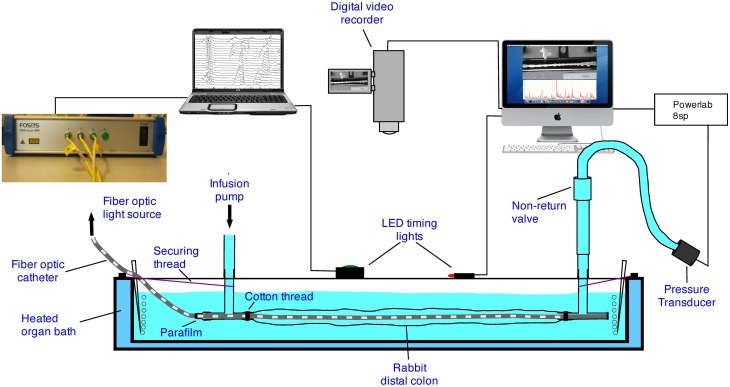
**The experimental setup.** In some of the preparations the fiber-optic catheter was replaced with an impedance and manometry catheter.

The experimental setup used in this preparation has been described in previous publications (Dinning et al., 2011, 2012a). The colonic segment was placed into an organ bath, with the oral end attached to a T-shaped plastic connector (Figure 1). The fiber-optic manometry catheter (or impedance manometry catheter) was placed through the same T piece and then inserted into the colonic segment (Figure 1). Parafilm wax (Benis Flexible packaging, Neenah, USA) was wrapped around the catheter and T piece to prevent the contents of the gut from leaking into the organ bath. The remaining arm of the oral T piece was connected to an infusion pump through which warmed (35°C) Krebs solution could be infused. The anal end of the colonic segment was attached to another T-shaped plastic connector. The catheter was gently pushed through the entire length of the segment of gut so that the catheter tip sat within the anal T piece (Figure 1). The open end of the anal T piece within the bath was sealed with Parafilm and the remaining arm of the anal T piece was connected to a closed-off outflow cannula. Pressures from the anal end were captured by a pressure transducer (P23ID, Gould USA), acquired in Chart 5.05 software via a PowerLab 8S recording system (ADI Australia). Both the oral and anal T pieces were fixed to the organ bath to prevent excessive shortening of the preparation.

### Fiber-optic manometry recording

Full description of the catheter design and validation can be found elsewhere (Arkwright et al., 2009a,b). The catheter, originally designed for *in vivo* studies in humans (Dinning et al., 2012b), incorporated 90 sensors spaced at 1 cm intervals. The catheter was attached to a spectral interrogator unit (FOS&S FBG-scan 804. FOS&S, Geel, Belgium) and pressures were recorded in real time using a custom written LabVIEW program (National Instruments, TX, US). The recorded manometric traces were viewed and analysed using software (PlotHRM) developed by one of the authors (LW). The software was written in Matlab (The MathWorks, MA, US) and Java (Sun Microsystems, CA, USA) (Dinning et al., 2012b).

### Impedance combined with manometry recordings

Intraluminal impedance was measured with a 3.2-mm diameter, solid-state manometric and impedance catheter incorporating twenty-five 1-cm–spaced pressure sensors and 12 adjoining impedance segments, each spaced at 2 cm (Uni-sensor USA Inc., Portsmouth, NH). Pressure and impedance data were acquired at 20 Hz (Solar GI Acquisition System, MMS, The Netherlands). These catheters have been extensively used in human esophageal manometry, including diagnosis of esophageal motility disorders (Omari et al., 2006, 2011).

### Video recording of diameter changes

**Figure 2:**
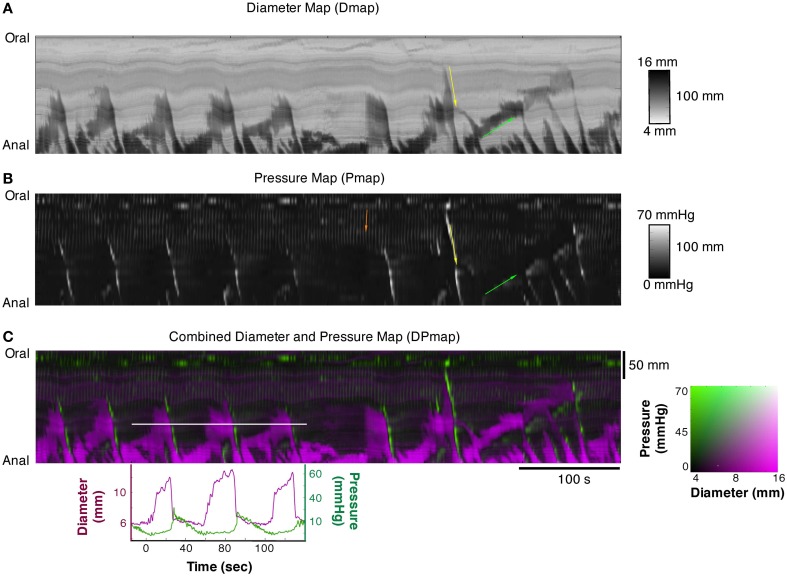
**(A)** Diameter Map (Dmap); **(B)** Pressure Map (PMap); and **(C)** composite Diameter/Pressure Map (DPMap). In the Dmap **(A)** the dilated regions are shown as dark areas. Peristaltic propagating contraction can be seen at regular intervals (yellow arrow) originating predominantly around the mid-point of the specimen. Slowly propagating antiperistaltic contraction are also apparent (Green arrow). In the Pmap **(B)** the propagating contractions appear as white oblique lines (yellow arrow) and the anti-peristaltic propagating contractions are highlighted by the green arrow. In the proximal regions of the colon, a series of vertical gray lines can be seen in the PMap (Orange arrow). These cyclic changes in the pressure profile, that are not apparent in the Dmap, represent myogenic rhythmic activity generated by slow waves. In the composite DPMap **(C)** changes in diameter are shown as shades of magenta, while pressure is shown in shades of green. Below the DPMap traces of diameter (magenta) and pressure (green) extracted along the white line in **(C)** are displayed and show the relation between changes in diameter with the corresponding changes in pressure. In this example there are three increases in diameter, each of which coincide with a liquid bolus entering the region. Associated with the movement of the liquid are peristaltic propagating contractions. As each of these moves into the region of the white line the diameter of the gut decreases and the pressure increases. The relations between pressure and diameter provide the basis for the orbits displayed in Figures 4, 5.

With either the fiber optic manometry catheter, or the impedance catheter in place, a digital video camera (Sony, DCR-TRV80E), positioned above the preparation (Figure 2), was used to record movies of colonic wall motion at video rate (iMovie, Apple, Cupertino, CA) with a Macintosh G4 computer, in clips of 10 min duration. These were then re-sampled down to 4 frames per second in Quicktime (Apple Inc., Cupertino, CA, USA).

### Experiment protocol

With either the fiber-optic or impedance-manometry catheter in the lumen, the gut was slowly distended (8 ml/min) by warmed (37°C) Krebs solution via the oral cannula. The maximal diameter corresponded to its normal diameter when full of feces. Recordings of video and manometry/impedance started within 20 min of the onset of distention. Manual, randomly activated, pulses were delivered to activate an LED in the field of view (Figure 1) to synchronize recordings. A ruler with marks at 1 cm intervals was placed next to the preparation to identify the locations of manometry sensors. Myogenic motor patterns were distinguished by blocking all neural activity with tetrodotoxin (0.6 μM) (Alomone Labs).

### Constructions of spatio-temporal maps

The videos of the rabbit colon were converted to spatio-temporal maps of changes in diameter (“Dmaps”) with software written in Matlab, adapted from the methods developed in our laboratory (Hennig et al., 1999). Briefly the diameter at each point along the preparation was calculated for each frame and converted into either a gray or color scale, to create a spatio-temporal map of diameter changes (DMaps—see Figure 2A). Another program (in Matlab) converted intraluminal pressures recorded by the fiber optic manometry catheter into spatio-temporal pressure map (“PMaps”), interpolating between points at 1 cm intervals (Figure 2B). Similar spatio-temporal maps were also constructed from the impedance traces (“IMaps”).

### Composite maps by aligning DMaps, PMaps, and IMap

Composite maps, combining PMaps with DMaps and/or IMaps, were then created in Matlab. These aligned maps enabled quantitative analysis of mechanical parameters during any given motor pattern. A color coded DPMap is shown in Figure 2C.

Spatial and temporal resolutions of DMaps, PMaps, and IMaps were adjusted to that of the lowest resolution method and baseline drift was removed from the pressure maps prior to alignment. Analytical routines in the software in Matlab were used to compare pressure or impedance with diameter during motility events at a single point on the preparation (Figure 2C). By plotting either pressure against diameter for single motility events, orbital plots were created that describe the relationship between these variables. From these orbits, we could deduce the state of excitation or inhibition of circular smooth muscle at every point along the preparation (see below). Such orbital plots have been extensively used in analysis of cardiac contractility (Covell and Ross, 2011). All measures were calculated assuming a circular cross-section of the colon.

### Principles of analysis of muscle contractility

Several mechanical states of the circular muscle of the wall of the intestine could be distinguished from combined maps and orbital plots

- actively contracting muscle, with contractions that were isometric, isotonic, or auxotonic
- actively relaxing muscle, either isometric, isotonic, or auxotonic
- passively shortening muscle, caused by a reduction in intraluminal pressure that reduces distension without active relaxation
- passively elongating muscle, associated with an increase in intraluminal pressure transmitted from another site along the preparation
- quiescent muscle which is showing no change in either diameter or pressure

Using software tools described above we could draw a line, of any length, across a composite DPMap and examine the orbit plots of Pressure/Diameter, (see Figure 4C) during single motility events, to identify the states of the muscle from second to second.

### Deducing the state of muscle from orbital plots

**Figure 3:**
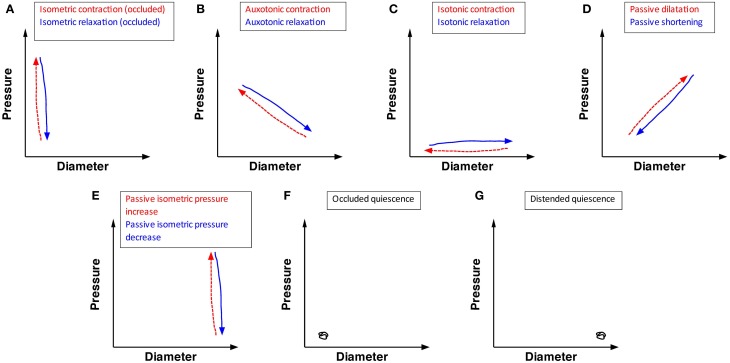
**The possible mechanical states of the circular muscle of the wall of the intestine, predicted on the bases of the relation between changes in pressure and diameter.** Twelve mechanical states are listed. **(A)** Isometric contraction (red hatched arrow) indicates an increase in pressure in an occluded region of gut (with no change in diameter). Isometric relaxation (blue arrow) indicates a drop in pressure with no change in diameter of the occluded gut. **(B)** Auxotonic contraction (red hatched arrow) indicates an increase in pressure with a corresponding decrease in diameter. Auxotonic relaxation (blue arrow) indicates a decrease in pressure with a corresponding increase in diameter. **(C)** Isotonic contraction (red hatch arrow) indicates a decrease in diameter with no change in pressure. Isotonic relaxation (blue arrow) indicates an increase in diameter with no change in pressure. **(D)** Passive dilation (red hatched arrow) indicates an increase in pressure with a corresponding increase in diameter. Passive shortening (blue arrow) indicates a decrease in diameter with a corresponding decrease in pressure. **(E)** Passive isometric pressure increase (red hatch arrow) indicated an increase in pressure in a distended region of gut (with no change in the gut diameter). Passive isometric pressure decrease (blue arrow) indicates a decrease in pressure in a distended region of gut (with no change in gut diameter). **(F)** Occluded quiescence indicates no change in pressure or diameter in an occluded region of gut. **(G)** Distended quiescence indicates no change in diameter and pressure in a distended region of gut.

The analysis concentrated on steady states of orbital plots (the near-linear components) as displayed in Figure 3. Five types of linear states could be distinguished in the orbital plots, from which three active conditions of the muscle could be identified, as follows:

1. *Isometric contractions* occur when the gut contracts against fixed or incompressible internal content (either the catheter, or the gut wall itself during lumen occluding contractions). This results in an increase in pressure with no associated change in diameter (vertical red hatched arrow, Figure 3A). When contractile activity stops, equivalent *isometric relaxations* occur in the occluded segment (vertical blue arrow Figure 3A).
2. *Auxotonic contractions* appear as oblique lines in the orbits indicating graded increases in pressure associated with graded decreases in diameter (oblique red hatched arrow Figure 3B). Likewise, when excitation is running down, or active inhibition occurs *auxotonic relaxations* occur with graded increases in diameter associated with graded reductions in pressure (oblique blue arrow Figure 3B).
3. *Isotonic contractions* appear as a decrease in diameter, associated with no increase in pressure (horizontal red hatched arrow Figure 3C) while an *isotonic relaxation* is shown as an increase in diameter with no change in pressure (horizontal blue arrow Figure 3C).

This analysis assumes that isometric, auxotonic, and isotonic contractions result from excitation of the muscle, either due to myogenic activity (slow-wave driven phasic contractions) or neurogenic activity, due to activity of enteric excitatory motor neurons (Box 1). It is less certain whether isotonic relaxations are active or passive. We have interpreted increases in diameter (elongations of circular muscle) that occurred in the absence of pressure changes as active isotonic relaxations, probably due to enteric inhibitory motor neuron input. Likewise, auxotonic relaxations are probably due to activation of enteric inhibitory neurons that reduce the tone.

In addition to the three active states of the muscle there are also two passive conditions;

1. Passive reductions of diameter (*passive shortening*) are represented by an oblique line indicating simultaneous graded reductions in pressure and diameter (oblique blue arrow Figure 3D), while *passive dilatations* are represented by parallel increase in both pressure and diameter (oblique red hatched arrow Figure 3D).
2. When the gut is fully distended by luminal content pushed against a closed end, a passive isometric increase in pressure can occur, signified by an increase in diameter with no change in pressure (*passive isometric pressure increase*; vertical red line Figure 3E). *Passive isometric pressure decrease* can also occur, pressure falls without a change in diameter (vertical blue arrow Figure 3E).

Finally two conditions of quiescence can occur in a segment of colon; which can be either empty (*occluded quiescence*) or full (*distended quiescence*), but no change in pressure or diameter (Figures 3F,G).

Between them, the 12 conditions illustrated in Figure 3 (five red hatched arrows, five blue arrows and two quiescent states) accounted for all of the linear components of orbital plots of motility events in the present study.

### Relation between velocity of shortening of circular muscle and intraluminal pressure

We also examined the rate of change of diameter versus pressure by examining non-linear segments of orbits. According to Hill's equation, maximal velocity of shortening occurs at low loads (isotonic contraction) and becomes minimal at high loads (Isometric contraction) (Box 2). The values of pressure versus diameters, extracted and calculated from the composite maps, were fitted to the Hill hyperbolic function:

(P+a)(V+b)=C

where *C* = (*P*_max_ + *a*) × *b*. We then followed the strategy of Wohlfart and Edman (1994) to identify the constants *a, b*, and *C*.

### Spatio-temporal maps derived from analysis of the linear section of orbital plots

In the final stage of analysis we converted DPMaps into spatiotemporal maps displaying each of the 12 possible mechanical states displayed in Figure 3 (five red arrows, five blue arrows and two quiescent states). The derivatives of diameter and pressure with respect to time were modeled as Gaussian distributions, as well as the boundary between occlusion and distention. The tail of each Gaussian on which a recorded sample fell was used to identify direction, i.e.; contraction or relaxation, in addition to the level of occlusion or distension. Using a Hidden Markov Model, this allowed classification into one of the twelve mechanical states (Rabiner, 1989). All areas undergoing active contraction and active relaxation were clustered, thus creating a map, which effectively displays all regions in which active, and passive muscle activity occur during myogenic and neurogenic motor patterns.

### Correlating impedance measurements and changes in diameter

In the course of this study, we also tested whether impedance measurements reliably reflect changes in internal diameter. To do this, we created combined impedance/diameter maps (IDMaps). Cross correlation analysis was performed in Matlab to establish the Pearson's correlation coefficient between measurements of external diameter and impedance changes. As the impedance catheter also contained pressure sensors we also created impedance/pressure maps (IPMaps) as well as DPMaps from the same segments of gut. This allowed us to compare the orbits created from the IPMaps to the orbits created from DPMaps at the same location in space and time.

## Results

The DMap of Figure 2A displays all of the known motor patterns of rabbit colon (Dinning et al., 2012a). Numerous examples of peristaltic (yellow arrow Figure 2A) and anti-peristaltic (green arrow Figure 2A) propagating contractions can be seen. These patterns are also reflected in the corresponding PMap (Figure 2B), where the increases in pressure indicate local changes in intraluminal pressure. A composite DPmap displaying both pressure and external diameter (Figure 2C) shows a number of oblique green streaks, reflecting increases in pressures propagating aborally. Each of these is preceded by increases in diameter (magenta areas), which occur ahead of the advancing pressure peak (e.g.: corresponding to the yellow arrow Figure 2B) reflecting accumulating propelled content filling an area where smooth muscle is probably inhibited. As shown by Dinning et al. (2012a) this pattern is abolished by tetrodotoxin, indicating that it represents neurally-dependent peristalsis. In these maps, small, rhythmic changes in pressure are seen particularly at the oral end of the preparation (orange arrow). They represent myogenic activity since they persisted after tetrodotoxin was added (see Figure 9; Dinning et al., 2012a).

### Mechanical state of the intestinal wall

**Figure 4:**
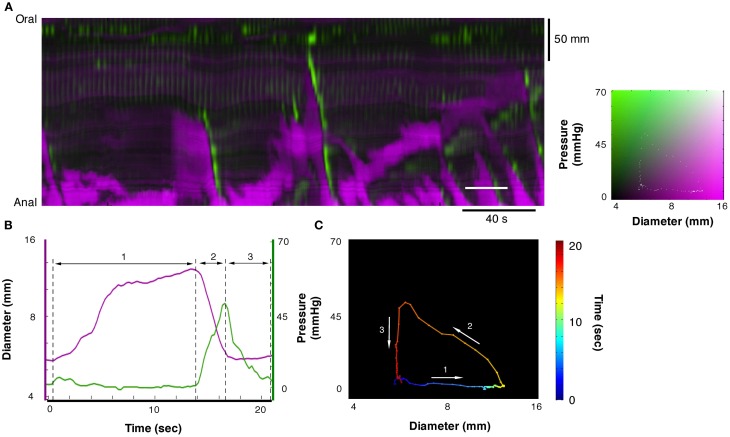
**A section of the DPMap shown in Figure 2C has been selected to demonstrate the variations in the mechanical states of the circular muscle.** The measurement for line plots, pressure, and diameter in **(B)** and the orbit plot in **(C)** have been taken from the white line in **(A)**. The orbit in **(C)** consists in a colored line, blue to red on the black background, and demonstrates the sequence of mechanical states (relationship between diameter and pressure) during the period of the white line in **(A)**. In this example the orbit consists in three linear segments with the direction each segment is traveling in the orbit shown by the white arrows. The first segment of the orbit, the blue to yellow indicates increasing diameter with no increase in pressure and corresponds to *isotonic dilatation* (see blue arrow in Figure 3C). The corresponding period of the diameter and pressure traces is shown in **(B)** as the first segment between the hatched lines. The next segment, yellow to orange, indicates decreasing diameter with increasing pressure, and corresponds to *auxotonic contraction* (see red hatched arrow **3B**). This segment is shown as the second region in **(B)**. The final segment, orange to red, indicates decrease in pressure with no change in diameter and corresponds to an *isometric relaxation* (section 3 in **B**; see also blue arrow in Figure 3A).

Each of the 12 mechanical states displayed in Figure 3 can be identified in the DPMap (Figure 2C). In Figure 4C, an orbital plot of pressure against diameter is shown (for the single event marked by the solid white line in Figure 4A). This reveals the dynamics of the intestinal muscle for the duration of the event. In Figure 4C, during phase 1, diameter initially increases with no change in pressure, as the colon accommodates liquid content propelled from a more proximal region. In phase 1, the orbit represents an *isotonic relaxation*. As a peristaltic contraction moves into the region of interest, there is a sharp change of direction in the orbital plot (into phase 2) and the gut diameter gradually decreases and pressure correspondingly gradually increases. This is an active *auxotonic contraction*. When the gut becomes fully occluded, there is another sharp turn in the orbital plot and phase 3 commences. Here pressure drops markedly, but there is no change in diameter, presumably because there is no fluid content to fill the relaxed region (*isometric relaxation*) (Figure 4C).

**Figure 5:**
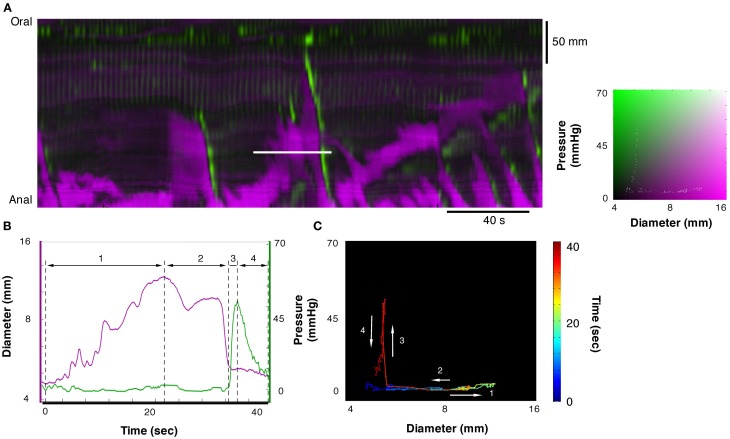
**Example of orbit plot taken from the DPMap (A).** The pressure and diameter line traces in **(B)** and the orbit in **(C)** are constructed from the white line in **(A)**. In this example the orbit consists of 4 segments. The first, blue to green section shows an *isotonic relaxation* (segment 1 in **B**; see also blue arrow in Figure 3C). The second segment, pale green to red, represents an *isotonic contraction*, where the gut diameter decreases with no change in pressure (segment 2 in **B**; see also red hatched arrow in Figure 3C). Next segment of the orbit straight up, indicates an *isometric contraction* (segment 3 in **B**; see also red hatched arrow in Figure 3A). Finally the gut relaxes, and the pressure drops, indicating *isometric relaxation* (section 4 in Figure 5B; see also blue arrow in Figure 3A).

In Figure 5C another orbital plot is shown for the region delimited by the solid white line. Again, the first linear phase (1 in Figure 5C) consists of *isotonic relaxation* as the gut begins to actively dilate to accommodate a bolus of liquid (with no change in pressure). In this instance, the peristaltic contraction starts suddenly (close to “1”) and diameter decreases with no change in pressure (*isotonic contraction*), presumably because of negligible downstream resistance to the movement of content. As the contraction occludes the lumen, diameter stops decreasing abruptly and pressure increases rapidly (vertical line at 3—*isometric contraction*). Eventually, the peristaltic contraction wears off, the squeeze is removed, pressure on the catheter drops but the gut remains occluded because there is no source of fluid content to re-fill it (*isometric relaxation*).

**Figure 6:**
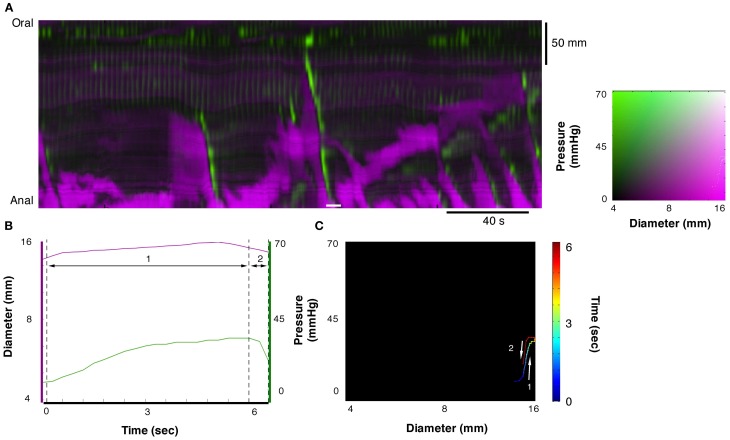
**Example of orbit plot at the anal end of the fully distended colon.** The measurement for line plots, pressure, and diameter in **(B)** and the orbit plot in **(C)** have been taken from the white line in the diameter/pressure spatiotemporal map in **(A)**. Segment 1 of the plot shows a period of increasing pressure with little change in diameter (*passive isometric pressure increase*). Segment 2 of the plot shows the reduction in pressure with little change in diameter (*passive isometric pressure decrease*). These two states are described in Figure 3E.

In Figure 6 an orbital plot is shown of a *passive isometric pressure increase* in a fully distended section that usually occurs at the anal end of the closed preparation. This distal region of the colon becomes distended by the bolus of liquid moving into the region traveling ahead of the peristaltic contraction. When the contraction reaches the distal end of the preparation the pressure within the region increases, but the already distended gut remains at a similar diameter (*passive isometric pressure increase*). When the contraction ends the pressure is released within the distended region resulting in decrease in the pressure with no reduction in diameter (*passive isometric pressure decrease*).

### Velocity of changes in diameter versus pressure

**Figure 7:**
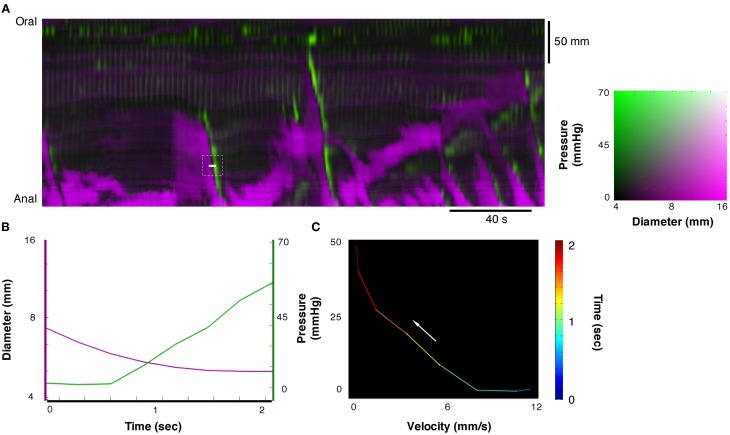
**From the composite DPMap (white line in A), traces of changes in diameter and pressure were extracted (B) from a short period.** During this period a contraction started as isotonic and ended as isometric **(C)**. Such orbit of dynamic mechanical state corresponds to the expected relation between force and length of muscles according to the Hill's model. Several such velocity/pressure segments were chosen in this composite DPMap to create the fitted Hill's equation in Figure 8.

**Figure 8:**
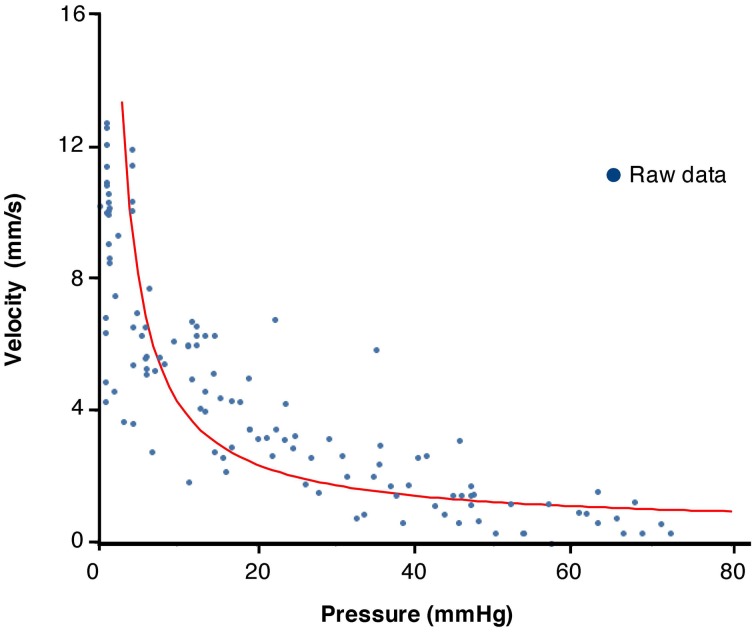
**Plot of values of velocity of contraction vs. pressure from 10 periods chosen from the composite DPMap in Figure 7 during isotonic to isometric contractions.** The plotted values were fitted by a rectangular hyperbole according to the method described in the method section. The curve shows the expected inverse relation between pressure and velocity of contraction described originally by Hill.

We tested whether the expected relation between force and velocity of contraction predicted by the Hill's equation, applies during physiological movements. A region of interest (white line Figure 6A) was chosen at the point where an isotonic relaxation transitions into isometric contraction; in this case the diameter decrease is associated with a pressure increase (Figure 7B). These data were then plotted as pressure against velocity of shortening (Figure 7B). We extracted eight examples of such orbits and Figure 8 shows the results with the Hill's rectangular hyperbolic function fitted according to the formula (*P* + *a*)(*V* + *b*) = (*P*_max_ + *a*) × *b* with the following values of the constants *a* = 0.037 HPa; *b* = 0.49 mm/s and *P*_max_ = 77 mmHg.

### Mapping discrete mechanical states

**Figure 9:**
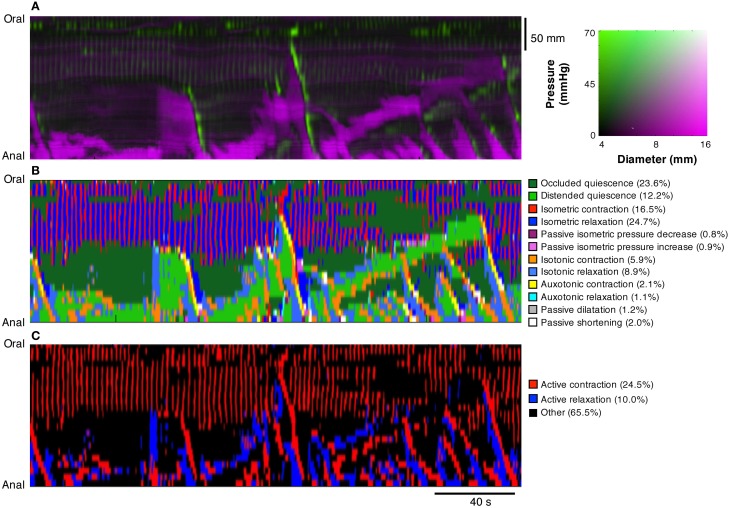
**Using the composite DPMaps (A) and extracted orbits constructed from the two variables (pressure and diameter), a spatio-temporal map of the steady states of the orbits (linear segments and quiescent orbits) was constructed (B).** In **(B)** each of the 12 possible mechanical states are mapped in different colors. The map portrays the periods of quiescence either when the intestine remains passively dilated (light green) or passively occluded (dark green). Red, orange, and yellow areas represent active contractions and mark the propagating area of contraction during neural peristalsis. Active relaxation (aqua and light blue) precedes both in time and space the propagating contraction. The regular myogenic activity in this example consists in isometric contractions and isometric relaxations. The last map **(C)** represents a simplification of the full composite map of states by clustering all areas undergoing active contraction (red) and, active relaxation (blue). All other passive states are in black. The spatio-temporal nature of active contractions and relaxations during neurogenic activity and myogenic activity is made very distinct.

Using the tools created in Matlab, orbital plots were created for each section of the composite DPmap shown in Figure 2C. From these orbits we extracted the linear sections that corresponded to specific functional states of the muscle and then plotted these on a new spatiotemporal map (Figure 9B). Thus, each of the 12 colors in this map represents one of the 12 defined mechanical state of the muscle.

To simplify the complexity of Figure 9B, we clustered isotonic, auxotonic, and isometric contractions and plotted them in red, thus revealing areas where the muscle is excited. Similarly we clustered isotonic and auxotonic relaxations and plotted them in blue to reveal areas where the muscle is inhibited. All other states were plotted in black (Figure 9C). Although we cannot exclude that some active states occur during these “black” periods, the areas in red and blue represent genuine active contracting and relaxing states of the muscle respectively. It should be noted that several long oblique red bands reflect peristaltic contractions propagating down the gut and that in each case these are preceded by downstream areas of inhibition in blue. Note also how the myogenic contractions (vertical red lines at the oral end of Figure 9C) are not associated with downstream or preceding inhibition.

**Figure 10:**
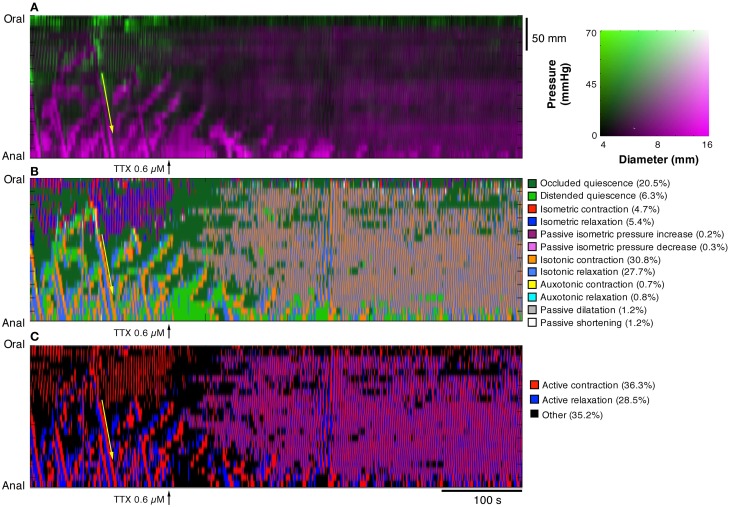
**After the addition of 0.6 μM tetrodotoxin (TTX) the map of steady state of the orbits (B, C), constructed from the DPMap (A), shows that the peristaltic propagating contractions (yellow arrow) are blocked, leaving the rhythmic myogenic activity.** Note that after TTX both myogenic contractions and relaxations become isotonic and appear both as active states.

In Figure 10, the effects of tetrodotoxin are shown. Tetrodotoxin rapidly blocked all peristaltic contractions (yellow arrows in Figures 10A,B), leaving the rhythmic myogenic activity (Figure 10A).

### Impedance measurements

**Figure 11:**
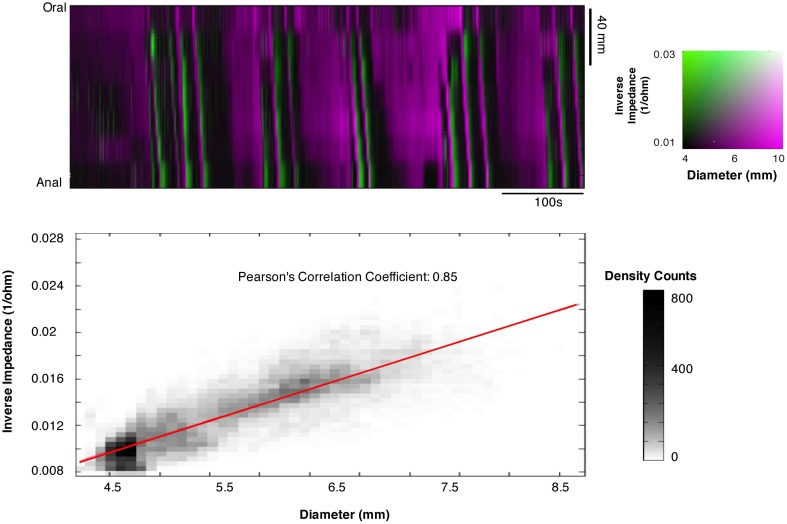
**Composite impedance/diameter map (IDMap).** The map displays the inverse impedance (green), which is proportional to internal cross sectional area of the gut and the diameters from video recording DMap (magenta). A strong correlation is shown between changes in impedance and changes in diameter with a Pearson's correlation coefficient of 0.85, indicating that the two measurements are equivalent.

**Figure 12:**
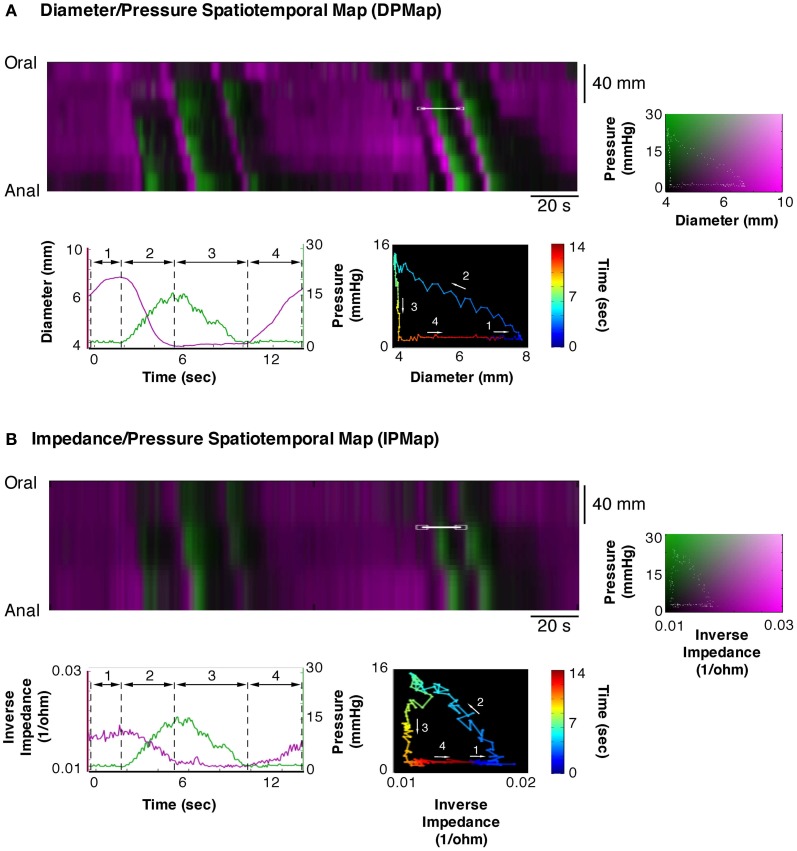
**Equivalent composite spatio temporal maps of diameter/pressure (DPMap; A) and impedance/pressure (IPMap; B).** Pressure is portrayed in green and diameter **(A)** and inverse of impedance **(B)** in magenta. As the impedance catheter also recorded pressure we are able to directly compared data generated from a DPMap with that generated from an IPMap. As can be seen here the extracted traces and orbits from both maps show the same four mechanical states and indicate the equivalence of the two methods of recording changes in diameter.

The Pearson correlation coefficient between changes of outer diameter measured from DMaps and the changes in impedance (inverse impedance is proportional to internal cross sectional area of the gut) over a 10 min recording, from a 12 cm segment of colon, was 0.85 (Figure 11). Orbital plots created from the DPMaps and IPMaps, at the same location in space and time, demonstrated remarkable similarity (Figure 12). Both of the orbital plots in Figure 12, show initial *isotonic relaxation*, followed by an *auxotonic contraction, isometric relaxation* and finally another *isotonic relaxation*.

## Discussion

Through the combination of spatiotemporal maps of gut diameter and intraluminal pressure we have demonstrated that it is possible to establish when and where active or passive contractions and relaxations of the intestinal muscle occur, and thereby deduced regions receiving input from enteric excitatory and inhibitory motor neurons. In addition, we have demonstrated very similar results from combinations of impedance and pressure, which indicate that this approach may be usefully applied to *in vivo* physiology in humans.

### Development of the method of analysis

The new method of analysis explored here was developed by combining methods used to study either intraluminal pressure (manometry) or diameter (video recordings). Spatio-temporal maps of intraluminal pressure (PMaps) and intestinal diameter have been successfully used separately in both human and/or experimental animals. The simultaneous development of Dmaps in several laboratories (Bouchoucha et al., 1999; Hennig et al., 1999; Bercik et al., 2000) led to accurate descriptions of the movements of isolated segments of intestine (D'Antona et al., 2001; Gwynne and Bornstein, 2007; Lentle et al., 2008, 2012; Hennig et al., 2010; Dinning et al., 2012a). In human clinical investigations, the development high-resolution spatiotemporal plots of pharyngeal and esophageal pressure (Williams et al., 2001) expanded the diagnostic capabilities of esophageal manometry (Pandolfino et al., 2008, 2009). More recently, impedance catheters (Fass et al., 1994; Omari et al., 2006) and fiber-optic manometry catheters (Arkwright et al., 2009a; Dinning et al., 2012b) have added to the clinical repertoire. However few studies have so far investigated what new insight can be obtained by combining the data from Dmaps and manometry (Dinning et al., 2011) or impedance and manometry (Omari et al., 2011).

In this study, we have developed composite maps to display diameter and pressure changes over time in the same plot (Figure 2C). This has provided new insight into the relationship between these two variables throughout significant lengths of gut, over periods of several minutes. However, developing changes in diameter and/or pressure are hard to read in these maps, limiting the value of simple visual assessment. A solution was found: to portray the relationship between diameter and pressure as orbital plots. This approach was originally developed for blood vessels, where it provided a more accurate description of the physics of the vascular wall than either recordings of diameter or pressure separately (Covell and Ross, 2011). In the colon, orbital plots revealed a deeper picture of the dynamic mechanical states of the muscle during physiological motor events. Critically, stiffness and/or compliance could be displayed: variables, which cannot be directly measured during motility because of the biomechanical complexity of the intestine's dynamic activity.

In this study, the 12 mechanical states, identified by extracting linear components from orbital plots, describe isometric, isotonic, and auxotonic shortening and lengthening under both active and passive conditions (see Figure 3). We identified examples of all these possible functional states during the complex motor activity in the isolated segments of colon. This suggests that, the mechanical state of the wall can be accurately attributed to active contractions or relaxations or to passive changes driven by propulsion of content from nearby active regions (Figures 4, 5).

Simplifying these 12-state maps to show just areas of muscle that are being excited and those that are being inhibited (Figures 9C, 10C), reflecting input from excitatory or inhibitory enteric motor circuits, also proved valuable. Through this simplification indirect means, we have established for the first time the spatio-temporal distribution of motor output by enteric neural pathways. This method also suggests that there are substantial intervening periods of localized quiescence of both neurons and muscle, during which only passive mechanical events occur.

Electrical recordings of inhibitory and excitatory neural inputs to the muscle in intact segments of intestine have only been reported in a few instances; under tightly controlled conditions (Yokoyama and North, 1983; Spencer and Smith, 2004). Combining our method with cellular recordings (Mazzuoli and Schemann, 2009) may allow us to directly test of the accuracy of our method of inferring the state of enteric circuits from biomechanical parameters.

### Description of motor patterns

In a previous work we described both myogenic and neurogenic motor patterns in the rabbit distal colon (Dinning et al., 2012a). Spontaneous myogenic motor activity of the circular muscle consisted of rhythmic myogenic “ripples” propagating in both directions, which were non-propulsive. Distension elicited anterogradely-propagating contractions of the circular muscle, which were highly propulsive (neural peristalsis), as well as retrograde circular muscle contractions (neural anti-peristalsis). These patterns of motor activity were also observed in the present study while recording with intraluminal catheters, suggesting that the presence of the catheters had little or no impact on overall motor activity.

The results of the present study have shown that peristaltic contractions are preceded by active relaxation of the muscle. This has previously been reported for propulsion of a semi-solid bolus (Bayliss and Starling, 1899; Costa and Furness, 1976) but this may be the first demonstration of dynamically polarized pathways in the propulsion of fluid by the colon.

The ability to identify and display which areas of intestine are excited, inhibited or quiescent will enable a much greater insight on the roles of neural and myogenic mechanisms underlying intestinal motility.

### Pressure/velocity relation of contractions during intestinal movements

The relation between velocity of shortening and tension of intestinal muscle has been investigated in isolated strip preparations of intestinal muscle (Gordon and Siegman, 1971a; Fung, 1993). They can be fitted by the hyperbolic function described by Hill in striated muscles (Hill, 1938). When we applied similar analysis (Wohlfart and Edman, 1994) to contractions extracted from DPMaps, we were also able to fit a rectangular hyperbolic function. This suggests that contractions in the moving intestine are a mixture of isotonic, auxotonic, and isometric and behave as predicted by basic muscle biomechanics. Circular muscle of the colon reached maximal shortening velocity when force (pressure) was minimal. As intraluminal pressure (load) increased, the peak rate of contraction decreased, eventually reaching zero at maximal intramural tension (Figures 7, 8). This corresponds to contractions starting isotonically with the intestine dilated and ending isometrically when the lumen is occluded.

### Potential applications to clinical studies in human

One of the major challenges facing clinical neurogastroenterologists will be to characterize the involvement of excitatory and inhibitory enteric motor pathways in motor activity in healthy human gut and how this is perturbed in disease. The present study has shown that video-based diameter maps invaluable, but can only be used in isolated preparations, and will therefore be of limited applicability in human intestine. Manometry has been often combined with fluoroscopy to overcome this limitation, allowing intraluminal pressure to be related to bolus transport (Massey et al., 1991; Ren et al., 1993). Such studies have been possible in the esophagus (Nicosia and Brasseur, 2002) because swallowing is under voluntary control, and therefore radiation exposure can be minimized. In other regions of the gut, prolonged exposure to X-rays would be required to capture the full range of motility patterns. An alternative strategy has been validated in the esophagus. Here, manometry has been combined with local measures of impedance during swallowing. Multichannel impedance has been used to measure the clearance of a bolus from the human pharynx and esophagus (Szczesniak et al., 2008, 2009), but can also be used to measure cross sectional area of the gastro-esophageal junction (McMahon et al., 2007; Hoppo et al., 2011), the upper esophageal sphincter (Omari et al., 2012; Regan et al., 2012) and the duodenum (Gregersen et al., 1990). In the present study, a combination manometry/impedance catheter was used to relate local diameter to intraluminal pressure. Our results showed excellent correlation between orbits calculated this way and with spatiotemporal DMaps and pressure (Pearson cross correlation of 0.85). We confirmed that impedance can be used to measure effectively the diameter of the lumen, with sufficient accuracy to allow analysis by our method of orbital plots. This may allow the current methods to be applied clinically to analyse motor defects in human patients with motility disorders, so long as the conductance of contents can be kept relatively constant to allow impedance measures of diameter. This may be possible in the esophagus or in the colon after bowel cleansing. Such a strategy might well give important new insights in the myogenic and neurogenic control of normal intestinal motility and unprecedented advantages in the diagnosis of motor disorders.

### Conflict of interest statement

The authors declare that the research was conducted in the absence of any commercial or financial relationships that could be construed as a potential conflict of interest.
